# COVID-19-associated mucormycosis: an update of anesthetic management

**DOI:** 10.1186/s42077-022-00216-3

**Published:** 2022-02-10

**Authors:** Nilesh Maganbhai Solanki, Rekha Nilesh Solanki, Arun Vrajlal Madaliya, Rasmita Haresh Jasoliya, Dhara Tushar Upadhyay

**Affiliations:** 1 Department of Anesthesia, B. J. Medical College and Civil Hospital, 44-Devshrusti Bungalows-II, B/h Kena Bungalows, Motera Stadium Road, Motera, Sabarmati, Asarwa, Ahmedabad, Gujarat 380005 India; 2 Department of Anesthesia, GCRI, B. J. Medical College and Civil Hospital, Asarwa, Ahmedabad, Gujarat India

**Keywords:** COVID-19, Diabetes mellitus, Fungal infection, Mucormycosis, Nasal endoscopic, Ophthalmic infection

## Abstract

**Background:**

Mucormycosis is uncommon, progressive fungal infection with mortality rate on higher side. The anesthetic management of surgical debridement in mucormycosis is challenging.

We evaluated the anesthetic management of mucormycosis patients associated with problems of airway management, predisposing factors, and adverse effect of antifungal therapy who underwent surgical resection of necrotized tissues.

**Results:**

Fifty-six patients presented with uncontrolled diabetes mellitus. All patients had history of COVID-19 infection and received steroid during treatment. Nasal endoscopic debridement was done in 43 patients, total maxillectomy in 14 patients. Temporalis flap was needed in five patients, and orbital exenteration was required in three patients. Central venous catheter was inserted in 17 patients, and invasive arterial monitoring was done in ten patients. All patients administered lyophilized amphotericin B (deoxycholate) in combination with surgical debridement.

Thirteen patients were shifted to ICU for further management and continuous hemodynamic monitoring. Fifteen patients were expired, and the mortality rate was 26.31%.

**Conclusions:**

Challenges include difficult intubation and renal function impairment due to prolonged antifungal therapy. Postoperative ICU management is important in mucormycosis patients due to comorbidities and rapid progressive infection after surgery.

## Background

COVID-19 is a relatively new diseases caused by severe acute respiratory syndrome coronavirus 2 (SARS-CoV-2). Bacterial and fungal co-infections may occur in COVID-19-infected patients due to presence of preexisting diseases like diabetes mellitus and lung pathology. Mucormycosis was first described by Fürbringer in Germany in 1876 (Fürbringer P 1876). It is caused by saprophytic fungi (Zygomycota, Phycomycota) of the order Mucorales. Rhino-orbital-cerebral mucormycosis (ROCM) is a rare and serious infection in COVID-19 patients. Patients with diabetics and concurrent use of steroids may be at higher risk for developing mucormycosis. The reason for sinusitis is inhalation of fungi spores via the nose, mouth, or mucosal laceration in the oronasal cavity. Rhinocerebral mucormycosis is divided into rhino-orbital, rhinomaxillary, and rhino-orbital-cerebral (Bavikar et al. 2017).

The main stay of treatment depends on early diagnosis, reversal of the patient’s underlying predisposing factors, intravenous antifungal drugs, and endoscopic surgical debridement of necrotizing tissue (Kulkarni et al. 2015).

The incidence of mucormycosis has significantly increased in diabetic patients, but the correct incidence/prevalence is not known due to less availability of population based studies (Bitar et al. 2009). In developing countries, rising trend of mucormycosis is due to diabetes mellitus, but hematological malignancies and transplantation are the most common underlying diseases in developed countries. The other important conditions that predispose to mucormycosis include prolonged neutropenia, corticosteroids, iron overload, trauma, and malnourishment (Petrikkos et al. 2012).

There is a study of 101 cases of COVID-19-associated mucormycosis (CAM), and 80/101 belonged to India and 19/101 from rest of the world (Singh AK et al. 2021). In a multicentric study of 21 patients of CAM in Egypt, the best outcome was observed in younger patients as compared to older patients and patients having cerebral extension (Alfishawy M et al. 2021).

The order Mucorales involves 261 species in 55 genera, but only 38 cause human infections (Walther et al. 2019).

It is challenging to diagnose of mucormycosis. There is a low sensitivity and specificity to diagnosis via clinical approach. Definitive diagnosis is based on histopathological examination of biopsy specimens from the involved area. For the extent of disease, imaging [computed tomography (CT scan) and magnetic resonance imaging (MRI)] are very important and useful tool (Skiada et al. 2020).

We present our experience of general anesthesia in 57 patients who underwent surgical debridement for rhino-orbital-cerebral mucormycosis.

## Methods

After obtaining local ethical committee approval (B.J.Medical College & Civil Hospital, Ahmedabad, Dated 19/01/2022, Ref No. EC/Approval/18/2022), written and informed consent was obtained from patients/relative. This prospective study was conducted in the ear nose throat (ENT) surgical operation theater in civil hospital, B.J. Medical College, from October 2020 to March 2021. There were 57 of either sex, American Society of Anesthesiologist physical status II–IV, age 32 to 80 years, weighing 52 to 120 kg, and patients who underwent surgery for rhino-orbital-cerebral (ROC) mucormycosis under general anesthesia.

### Inclusion criteria

All cases were admitted to civil hospital between October 2020 and March 2021 with histological/mycological diagnosis of mucormycosis affecting any organ.

### Exclusion criteria

1. All cases diagnosed with mucormycosis with any hematological malignancy
2. Post chemotherapy status
3. Post radiotherapy status or any solid organ carcinoma
4. Post organ transplant recipients

### Clinical presentation

The early stage of sinus mucormycosis may be associated with nasal congestion, nasal discharge, fever, headache, and malaise and sinus tenderness. When the diseases progress, involvement of ethmoidal and maxillary sinus may lead to tissue infarction with necrosis of turbinate and palatal necrosis. There may be facial or periorbital swelling, blurred vision, ptosis, proptosis, severe pain, and loss of vision which are indicative of involvement of orbital nerve and vessels.

### Medical management

Aggressive medical therapy for underlying predisposing factors and prompt initiation of antifungal drugs is recommended. The first-line medical treatment is systemic administration of lyophilized amphotericin B (deoxycholate) 1 mg/kg intravenously for every 24 h. With the requirement of high doses, nephrotoxicity may result. However, intravenous liposomal amphotericin B 10 mg/kg for every 24 h is protective for renal function. Both drugs destroy the cell wall of the fungus. In some cases, combination of amphotericin B and oral posaconazole 300 mg every 24 h may be given which inhibits the growth of the fungus. Because systemic antifungal drugs are not able to reach the infected tissue due to vascular thrombosis, surgery should be considered as early as possible.

### Surgical management

The surgical procedures, both endoscopic and open approaches, are utilized for debridement of all necrotic tissue. These techniques include medial or radical ethmoidectomy, maxillectomy, and sphenoidectomy. In mucormycosis, the involved tissue does not bleed, so the surgeon does debridement until bleeding tissue is encountered. Orbital exenteration is to be done in extensive spread of diseases. Teamwork from otorhinolaryngology surgeon, oral and faciomaxillary dental surgeon, ophthalmologist, and skilled anesthesiologist is required with good communication and in-depth knowledge in caring of these patients.

### Preoperative assessment and preparation

Pre-anesthetic evaluation revealed history, physical examination, necessary laboratory and radiological finding, respiratory and cardiac evaluation, optimization of predisposing factors, and airway management.

### Anesthetic management

General anesthesia was given by experienced anesthesiologist for all the surgical procedures for mucormycosis. In the operation theater, two large bore intravenous lines were secured and pulse oximetry (SpO_2_), electrocardiography (ECG), heart rate (HR), non-invasive blood pressure (NIBP), and EtCO_2_,were monitored for each patient.

A difficult intubation cart was kept ready. All patients were preoxygenated with 100% oxygen (3–5 L/min) for 3 min. All patients were premedicated with inj. glycopyrrolate (0.004 mg/kg), inj. ondansetron (0.15 mg/kg), inj. midazolam (1 mg), and inj. fentanyl (1–2 μg/kg) intravenously. Induction of anesthesia was done with intravenous propofol (1–2 mg/kg) or etomidate (0.2–0.6 mg/kg) intravenously according to body weight of patients. To attenuate stress response due to laryngoscopy, intravenous xylocard 1.5 mg/kg was given. Tracheal intubation was facilitated by intravenous succinylcholine 2 mg/kg. Each patient was intubated with adequate size oral cuffed endotracheal tube, and endotracheal tube was fixed on the left side of the angle of mouth after confirmation of equal bilateral air entry on the chest and EtCO_2_ value monitoring continuously. Oral packing was done in all patients to prevent aspiration of blood due to surgical procedure. All patients were ventilated mechanically with Drager Fabius plus (Drager, Germany) at tidal volume of 6 ml/kg of predicted body weight with frequency of 10–12 breaths per minute. Heart rate, NIBP, SpO_2_, EtCO_2_, and temperature were noted at a 15-min period during preoperatively and every hourly postoperatively. The EtCO_2_ value was maintained within 35–45 mmHg by adjusting tidal volume and ventilatory rate. Maintenance of anesthesia was done with sevoflurane (0.6–2.0%) or desflurane (2–6%) in mixture of oxygen (50%) and air (50%). Bolus dose of intravenous atracurium (0.5 mg/kg) with incremental dose (0.1 mg/kg) was administered as per requirement. During surgery, adequate fluid balance with intravenous normal saline and ringer lactate administered @ 10–15 ml/kg/h. The transfusion of blood products were given as per requirement. All patients were reversed by using intravenous glycopyrrolate (0.008 mg/kg) and neostigmine (0.05 mg/kg) after completion of surgery. Patients with adequate spontaneous respiration (respiratory rate > 14/min, tidal volume > 6 ml/kg), SpO_2_ > 95% on air, and who follow verbal commands were extubated after oral suction and removal of oral pack. All patients were transferred to the postoperative ward for careful observation. However, 13 patients were transferred to the ICU with endotracheal tube in situ in 5 patients for further management.

### Postoperative management

After completion of surgery, inj. tramadol (0.5–1 mg/kg) or inj. paracetamol 1.0 g was given intravenously for postoperative pain relief.

### Procedural data

The type of collection data was done by observational method. Demographic data was obtained from patient’s indoor case and preoperative history, examination, and laboratory findings.

Duration of surgery was defined as time from the initiation of the surgery to the end of the surgery. Duration of anesthesia was defined as time between inductions of anesthesia to the shift of the patient to postoperative care ward. Duration of ICU stay was defined as time between arrivals in ICU to the shift of the patient to the ward. Hospital stay, discharge, and mortality rates were also recorded.

### Statistical analysis

In this study, statistical calculations are carried out using Microsoft Office Excel 2010. Collected data were presented as mean, numbers, and percentages as appropriate. Quantitative variables were analyzed by using mean and range. Categorical variables were measured as frequencies and percentages.

## Results

Fifty-seven COVID-19-associated mucormycosis adult patients underwent surgical debridement. Demographic details like age, weight, sex, and predisposing factors are presented in Table 1.

**Table 1:** Demographic characteristics

Variables	Patients number
Age (years)	60 (32–80)
Sex (male/female)	40/17
Weight (kg)	67 (52–120)
ASA* grade II/III/IV	18/13/26
Mallampati I/II/III	24/29/4
Post procedure elective tracheostomy	1/57
**Predisposing factors**	
Diabetes mellitus	24
DM* + HTN* + IHD*	15
DM + HTN	17
HTN	1
CABG*	2
Angioplasty	3
On steroids	57
COVID-19-associated mucormycosis	57
History of ICU* admission during COVID-19 infection	19
History of requirement oxygen (ICU/ward) during COVID-19 infection	28
Average day stay in ward/ICU during COVID-19	11 (6–25)
Occurrence of mucormycosis average days after COVID-19 infection	32 (6–150)

Data presented as mean, average, or number

There were 40 (70.12%) male and 17 (29.82%) female patients with a mean age of 60 (range 32–85) years. Mean body weight was 67 (range 58–120) kg. Eighteen patients belonged to ASA grade II, 13 patients to ASA grade III, and 26 patients to ASA grade IV. Total 56 (98.24%) patients presented with uncontrolled diabetes mellitus (high blood sugar and on insulin treatment). Among comorbid patients, history of CABG was noted in two patients and angioplasty also in three patients. All patients had received steroid [intravenous dexamethasone (6 mg per day for a maximum 10 days) and, in moderate cases, intravenous methylprednisolone (0.5–1 mg/kg/day for 3 days)] during COVID-19 treatment. Nineteen patients were needed ICU admission during COVID-19 infection. Twenty-eight patients had history of requirement of oxygen during COVID-19 infection (ICU/ward stay). The occurrence of mucormycosis, average days after COVID-19 infection, was 32 (6–150) days.

Thirty-eight patients had a diagnosis of rhino-orbital, while 15 patients had rhinomaxillary and four patients had a diagnosis of rhino-orbital cerebral mucormycosis (Table 2).

**Table 2:** Types of mucormycosis

Types	Number
Rhino-orbital	38 (66.6%)
Rhino maxillary	15 (26.3%)
Rhino-orbital-cerebral	4 (7.1%)
Pulmonary	0
Others	0

Data presented as number (%)

Nasal endoscopic debridement was performed in 43 patients, and maxillectomy was done in 14 patients. Temporalis flap was needed in five patients, and three patients required orbital exenteration (Table 3).

**Table 3:** Diagnosis and surgical procedures

Area involved (CT scan/MRI)	Surgical procedure	Number
Nose, ethmoid, maxilla, choncha, turbinates	Nasal endoscopic (NE) debridement	43 (75.44%)
Maxilla, ethmoid, hard palate, floor of orbit	Maxillectomy	14 (24.46%))
Maxillary, palate, intraorbital, intracerebral cellulitis, cavernous sinus thrombosis	Orbital exenteration/temporalis flap	3/5

Data presented as number (%)

All patients were administered crystalloids, while 5 patients needed colloids additionally and 6 patients were given blood for replacement of blood loss. Elective vasodilator therapy (nitroglycerine infusion) was started in 12 (21.05%) patients due to cardiac comorbidities. Seven patients required ionotropic support (noradrenaline infusion) during perioperative period. Invasive arterial blood pressure monitoring was done in 10 (17.54%) patients, and central venous catheterization was needed in 17 (29.82%) patients. Urinary catheterization was done in 15 patients. Fourteen patients required nasogastric (Ryle’s) tube insertion postoperatively. Mean duration of surgery was 170 (range 75–300) min. Mean duration of anesthesia was 180 (range 85–315) min (Table 4).

**Table 4:** Procedure data

Name	Number
Arterial cannulation	10
Central venous catheterization	17
Urinary catheterization	15
Nasogastric Ryle’s tube insertion	14
Total crystalloids, ml	1500 (400–3500)
Total colloids, ml	500 (0–500)
Total blood/blood products, ml	600 (0–600)
Urine output	300 (90–1000)
Duration of surgery (min)	170 (75–300)
Duration of anesthesia (min)	180 (85–315)

Data presented as mean, average, or number

Fifty-two patients were extubated in the operating room, and five patients were shifted to the ICU with endotracheal tube in situ. Total 13 patients had required ICU admission for further management, and five patients needed mechanical ventilator support. A total of 33 patients were admitted in the ward for more than 25 days. All patients were given lyophilized amphotericin B (deoxycholate) as antifungal therapy, while three patients received additional oral posaconazole. Forty-two patients were discharged, and death occurred in 15 (26.31%) patients (Table 5).

**Table 5:** Postoperative recovery

	Days	Number
Extubation in operating room	Yes	52
	No	5
Shifted to postoperative ward		44
Shifted to ICU		13
Postoperative ICU stay duration (days)	2	7
	3	2
	4	2
	≥ 5–10	2
Postoperative ventilator duration (days)	1	4
	≥ 4–6	1
Hospital stay ward (days)	≥ 15	4
	≥ 20	5
	≥ 25	30
	≥ 30–40	3
Discharged	15–40	42
Expired	3–15	15/57
Mortality rate		26.31%
Intravenous amphotericin B continue, day	14 (8–45)	57

Data presented as number (%)

Forty-two patients had difficulty in mask ventilation, 29 patients were intubated with the help of intubating stylet, and 14 patients were intubated with help of bougie (Table 6).

**Table 6:** Problem during perioperative anesthesia management

Name	Number
Difficult mask ventilation	42 (73.68%)
Intubation with help of stylet	29 (50.87%)
Intubation with help of bougie	14 (24.56%)
Preoperative correction of hypokalemia	5 (8.77%)
Preoperative elevated serum creatinine	7 (12.28%)
Nitroglycerine infusion	12/57
Noradrenaline infusion	7/57
Hemodynamic instability during surgery	19/57

Data presented as number (%)

## Discussion

The rise and spread of COVID-19 across the world lead to a pandemic situation.

Secondary infection may occur in COVID-19-infected patients due to underlying diseases, such as diabetes mellitus, use of immunosuppressive therapy, previous lung infection, and risk of hospital acquired infection which may lead to morbidity and mortality (Chen et al. 2020).

COVID-19 is associated with secondary infection such as bacterial and fungal due to immune dysregulation and also exacerbation of pre-existing fungal diseases because of widespread use of steroid/monoclonal antibodies/broad spectrum antibiotics (Mehta et al. 2020).

According to guidelines, COVID-19 patients who are on ventilator or requirement of oxygen, dexamethasone (6 mg per day for a maximum 10 days) (Recovery group 2020) is given, and, in moderate cases, intravenous methylprednisolone 0.5–1 mg/kg/day is given for 3 days, and in severe cases, it can be used 1–2 mg/kg/day (mohfw.gov.in 2020).

In our study, all patients had received dexamethasone/methyl prednisolone during treatment of COVID-19 infection and developed mucormycosis.

Mucormycosis is uncommon and rapidly progressive infection which is caused by saprophytic fungi (Zygomycota, Phycomycota) of the order Mucorales. Ninety percent of the cases of rhinocerebral mucormycosis is caused by *Rhizopus* (Wali et al. 2012). The most important predisposing factors to mucormycosis include uncontrolled diabetes mellitus with or without ketoacidosis, prolonged use of corticosteroids, malnutrition, hematological malignancies, and severe neutropenia (Petrikkos et al. 2012). The different types of mucormycosis are rhinocerebral, pulmonary, gastrointestinal, and central nervous system and may involve different body parts.

In the early stage, infected nasal mucosa which may appear normal becomes edematous followed by black necrotic tissue. The first clinical signs in rhinocerebral mucormycosis are presented with headache, facial swelling, pain, black eschar nasal discharge, and periorbital edema. In the extensive phase, orbital structures involvement may lead to proptosis, ptosis, and loss of vision (Wali et al. 2012).

Rhino-orbital-cerebral mucormycosis first occurs in the nasal or oral mucosa, involving the paranasal sinuses, and then spread to the orbit via ethmoid and maxillary sinuses or via nasolacrimal duct (Abramson et al. 1984). Intracerebral involvement may occur from orbit via orbital apex, orbital vessels, or via cribriform plate (Abramson et al. 1984).

In our study, all patients were presented with headache, facial swelling, pain, black eschar nasal discharge, and periorbital edema. Loss of vision and proptosis were present in 5 patients, who involved orbital structure. Two patients had facial nerve palsy.

Mucormycosis is aggressive, and fatal outcome may occur in uncontrolled diabetic patients when it is combined with ketoacidosis, where ketone bodies displace the iron bound to serum proteins and thus increased availability of free iron which causes growth of fungi, impairing phagocytic function as well as reducing the efficiency of chemotaxis (Bavikar et al. 2017; Wali et al. 2012; Pinto et al. 2011). Because of higher affinity of fungus to arteries, it adheres to its walls, and growth along their internal elastic lamina occurs which may result in thrombosis, ischemia, and necrosis of surrounding tissues.

In a study, it was revealed that 88.2% of rhinocerebral mucormycosis had occurred in uncontrolled diabetes mellitus, and 53.3% of them had ketoacidosis (Wali et al. 2012).

In our study, 56 (98.24%) patients presented with uncontrolled DM, and out of them, 14 (24.56%) patients had ketoacidosis also.

A treatment strategy should start with elimination of associated diseases and optimization of the patient’s condition. Systemic broad spectrum anti-bacterial and antifungal are started for control of infection and insulin to control blood sugar.

Antifungal therapy with lyophilized amphotericin B is nephrotoxic, and electrolyte imbalance (hypokalemia, hypomagnesemia) and hypotension may occur (Karaaslan et al. 2019).

In our study, all patients received lyophilized amphotericin B (deoxycholate). However, seven patients developed increase level of creatinine, and amphotericin B was on hold for some days. Five patients had developed hypokalemia (K^+^ < 3.0), and correction was done with KCL drip with close monitoring. The duration of antifungal therapy was with a mean of 14 (8–45) days and was individualized to every case.

However, liposomal amphotericin B can be used for longer period due to less nephrotoxic (Cagatay et al. 2001; Karaaslan et al. 2019). A study reported that administration of retro bulbar injection of amphotericin B may be a therapeutic option in cases of rhino-orbital mucormycosis where aggressive orbital exenteration is not favored (Hirabayashi et al. 2017).

Treatment of mucormycosis is aggressive surgical debridement, and the procedure lasts longer. It may result in cosmetic deformity which leads to severe anxiety in patients. So, preoperative anxiolytic plays an important role for perioperative anesthetic management of the patients (Wali et al. 2012).

In our study, all patients were premedicated with inj. midazolam (1 mg) intravenously preoperatively.

Patients with rhino-orbital cerebral (ROC) mucormycosis may experience difficult mask ventilation due to pain and facial edema associated with fungal debris (Karaaslan et al. 2019).

In our study, mask ventilation was difficult in 42 patients after induction due to diffuse fungal debris and facial edema but was managed with oral airway.

We considered that the operation theater has proper sized facemask, intubating stylets, gum elastic bougie, supraglottic device, video laryngoscopes, rigid laryngoscope with blades of different sizes, and emergency tracheostomy for unexpected difficult airway management.

There is no documentation for ideal intravenous anesthetic agent for induction in mucormycosis patients. In vitro studies suggested that volatile anesthetic agents inhibited the growth of fungal cell (Karaaslan et al. 2019).

In our study, propofol was the first choice of induction agent, but in cardiac patients with mucormycosis, we used etomidate as an induction agent. Sevoflurane/desflurane was used as a volatile anesthetic agent during maintenance of anesthesia.

Eckmann et al. reported that they could not succeed to intubate the patient due to supraglottic edema and inserted a tracheostomy tube (Eckmann et al. 1998).

In our study, 29 patients were intubated with the help of intubating stylet, and 14 patients required bougie for intubation. Only one patient had required elective tracheostomy after completion of the surgery.

Mucormycosis patients are in high risk of hemodynamic instability and fluid-electrolyte imbalance. Some patients may require blood and blood product transfusion, fluid replacement, and prolonged ICU stay, which may necessitate central venous cannulation (Karaaslan et al. 2019).

Patients with mucormycosis are more prone to develop hypotension during perioperative period. It is challenging to maintain adequate renal perfusion and to prevent progression of renal damage in mucormycosis patients. Therefore, arterial cannulation is required for continuous arterial blood pressure monitoring and repeated arterial blood gas sampling during perioperative period (Karaaslan et al. 2019).

In our study, central venous catheter was inserted via internal jugular vein in five patients and via femoral vein in 12 patients. Elective vasodilator (nitroglycerine infusion) therapy was started perioperatively in 12 patients due to comorbidities. Seven patients had developed severe hypotension and required ionotropic (noradrenaline infusion) support.

In our study, invasive arterial cannulation was performed in ten patients. Sample for arterial blood gas analysis was sent perioperatively and when indicated.

Mucormycosis has angioinvasive ability and may result in thrombosis of the blood vessel leading to necrosis and ischemia of surrounding tissues (Yohai et al. 1994). So, the primary goal of the surgery is to debride all necrotic tissues.

In our study, the primary goal was to debride necrotic tissues by nasal endoscopic procedure in 43 patients. Fourteen patients needed maxillectomy. On the other hand, orbital exenteration can be lifesaving in the extensive stage of acute fungal infection (Blitzer et al. 1980). Orbital exenteration was done in three patients in our study.

Temporalis flap is preferred for primary reconstruction to close the defect after orbital exenteration. It results in a better cosmetic appearance due to it having better vascularization than dermal and skin grafts (Uvar et al. 2015). In our study, temporalis flap was done in a total of five patients.

Literature shows that there are increased morbidity and mortality due to septicemia, multiple organ failure, immunosuppression, and absolute neutropenia (Elihu et al. 2007).

Hyperbaric oxygen therapy is a more useful adjunctive treatment in mucormycosis with diabetic patients with a survival rate of up to 94%. It is fungicidal, improves neutrophil activity, inhibits growth of Mucorales, and improves flow of oxygen to ischemic tissues and wound healing by releasing growth factors (John et al. 2005).

The patients are diagnosed at the late stages of the diseases with an extensive involvement of the sinuses as well as increased serum creatinine level and thrombocytopenia resulting in high mortality rate up to 90% (Jeong et al. 2015). There is a study showing that the benefit of aggressive medical along with surgical treatment in rhinocerebral mucormycosis patients has recently decreased mortality rate from 88 to 21% (Elihu et al. 2007). The mortality in CAM (87.5% in the current series) may be even higher than that observed in non-COVID patients (Garg et al. 2021).

In our study, all patients received both surgical debridement of necrotized tissue and antifungal therapy. In our study, death occurred in 15 patients between 3 and 15 days after surgery. The mortality rate of our patients was 26.31% (15/57) due to early diagnosis as well as aggressive medical and surgical management. However, seven patients had elevated level of creatinine, and five patients had developed thrombocytopenia.

Postoperative ICU care is very essential due to the presence of co-existing diseases and rapid progression of infection after surgery. In our study, 13 patients had required ICU admission for further management.

A limitation of our study was the non-availability of hyperbaric oxygen therapy in our institute. We did not use liposomal amphotericin B, as we had large number of cases and liposomal amphotericin B is an expensive drug.

## Conclusions

Rhino-orbital-cerebral mucormycosis is a rare fungal disease, a big threat in uncontrolled diabetic and immunocompromised patients. Challenges are difficult intubation and renal function impairment due to prolonged antifungal therapy. Poor prognosis is due to delay in diagnosis and treatment. Standard treatments are multidisciplinary approach including reversal of predisposing factors, immediate initiation of antifungal therapy, and surgical debridement of necrotized tissues. Postoperative ICU management is paramount in mucormycosis patients because of comorbidities and rapid progressive infection after surgery.

## Data Availability

The data used/analyzed during this study are available from corresponding author on reasonable request.

## Abbreviations

COVID-19: Coronavirus disease 2019
ASA: American Society of Anesthesiology
SARS: CoV-2-severe acute respiratory syndrome coronavirus 2
ROCM: Rhino-orbital-cerebral mucormycosis
CAM: COVID-19-associated mucormycosis
CT scan: Computed tomography
MRI: Magnetic resonance imaging
ENT: Ear, nose, and throat
SpO_2_: Pulse oximetry
ECG: Electrocardiography
HR: Heart rate
NIBP: Non-invasive blood pressure
EtCO_2_: End-tidal carbon dioxide
inj.: Injection
@: At the rate
KCL: Potassium chloride
ROC: Rhino-orbital-cerebral
DM: Diabetes mellitus
HTN: Hypertension
IHD: Ischemic heart diseases
CABG: Coronary artery bypass graft
ICU: Intensive care unit
NE: Nasal endoscopic
